# Phytosynthesis and characterization of tin-oxide nanoparticles (SnO_2_-NPs) from *Croton macrostachyus* leaf extract and its application under visible light photocatalytic activities

**DOI:** 10.1038/s41598-024-60633-2

**Published:** 2024-05-11

**Authors:** Yonas Etafa Tasisa, Tridib Kumar Sarma, Tarun Kumar Sahu, Ramaswamy Krishnaraj

**Affiliations:** 1 Department of Physics, College of Natural and Computational Sciences, Wollega University, Nekemte, Ethiopia; 2 Department of Chemistry, Indian Institute of Technology Indore, Indore, 453552 Madhya Pradesh India; 3 Department of Mechanical Engineering, College of Engineering and Technology, Dambi Dollo University, Dembi Dolo, Ethiopia; 4 Center for Global Health Research, Saveetha Institute of Medical and Technical Sciences, Saveetha University, Chennai, India

**Keywords:** *Croton macrostachyus*, Characterization, SnO_2_, Visible light, Nanoparticles (NPs), Green synthesis, Chemistry, Engineering, Materials science, Nanoscience and technology, Physics

## Abstract

Nanotechnology is rapidly becoming more and more important in today's technological world as the need for industry increases with human well-being. In this study, we synthesized SnO_2_ nanoparticles (NPs) using an environmentally friendly method or green method from *Croton macrostachyus* leaf extract, leading to the transformation of UV absorbance to visible absorbance by reducing the band gap energy. The products underwent UV, FTIR, XRD, SEM, EDX, XPS, BET, and DLS for characterization. Characterization via UV–Vis spectroscopy confirmed the shift in absorbance towards the visible spectrum, indicating the potential for enhanced photocatalytic activity under visible light irradiation. The energy band gap for as-synthesized nanoparticles was 3.03 eV, 2.71 eV, 2.61 eV, and 2.41 eV for the 1:1, 1:2, 1:3, and 1:4 sample ratios, respectively. The average crystal size of 32.18 nm and very fine flakes with tiny agglomerate structures of nanoparticles was obtained. The photocatalytic activity of the green-synthesized SnO_2_ nanoparticles was explored under visible light irradiation for the degradation of rhodamine B (RhB) and methylene blue (MB), which were widespread fabric pollutants. It was finally confirmed that the prepared NPs were actively used for photocatalytic degradation. Our results suggest the promising application of these green-synthesized SnO_2_ NPs as efficient photocatalysts for environmental remediation with low energy consumption compared to other light-driven processes. The radical scavenging experiment proved that hydroxyl radicals (^_^OH) are the predominant species in the reaction kinetics of both pollutant dyes under visible light degradation.

## Introduction

The term nanostructured materials (NsM) refers to microstructured substances whose characteristic length scale is on the order of a few nanometers (usually 1–10 nm)^1^. The definition of nanotechnology is the production of material using precise chemical or physical procedures to produce substances with certain characteristics that are capable of being used in certain purposes. Since nanomaterials possess their low effect, high specific surface area, and distinctive configuration characteristics, nanometal oxides are a type of nanomaterial with a broad variety of applications^2^.

The two categories of methods developed for the synthesis of NsM are as follows. Top-down and bottom-up synthesis techniques. In the top-down method, NsMs are assembled from pre-made or pre-existing structural constituents (such as supramolecular units, nanometer-sized crystals, etc.). These components merge to form a solid with a nanometer-scale microstructure. In bottom-up approaches, complex structures or materials are built from simpler components or starting materials. In the context of green synthesis, this often involves the assembly of nanomaterials, nanoparticles, or other structures from environmentally friendly and sustainable resources. Examples of this method of synthesis are evaporation on a cold substrate or crystallization from the glassy state^3^. Chemical precipitation, solvothermal synthesis, gel sol, chemical vapor deposition, and photochemical oxidation are examples of conventional nanoparticle manufacturing techniques, which comprise high fabrication costs, adverse effects on humans or the environment, require high-risk chemicals, increasing global warming and are complex to scale up. Due to this reason, unconventional techniques such as green synthesis are recently used^4,5^. By using processes that are more respectful of the environment, living things, and nonliving things present in nature, such as bacteria, fungi, and plants, green chemistry creates new materials. These synthetic processes have a beneficial effect on the protection of the environment. They are also unique in that they have a simple, non-toxic process, have no specific or complex requirements, and are faster, safer, and cheaper than conventional methods^6^.

Studies have been reported in the scientific community on the green production of various NPs, including Gold, silver, palladium(Pd), Magnesium oxide, copper oxide, Zinc oxide, Titanium oxide, Iron oxide, Tungsten trioxide(WO_3_), tin oxide, etc. Some n-type semiconductors like SnO_2_ have a critical energy gap of 3.56 eV at 300 K discern it to be widely used in many applications including gas sensors, Li-particle batteries, optoelectronic devices, and anti-reflective coatings, it is increasingly acknowledged for its pleasantly high conductivity, simplicity, safety and gas compliance^7,8^.

Nanostructured metal oxide semiconductors have the potential to entirely remove dangerous compounds from industrial wastewater because of their distinctive optical, physicochemical and electronic properties. Due to their extensive use in many fields such as textiles, cosmetics, pharmaceutical, and food industries, dyes and aromatic compounds are attracting more and more attention^9^.

Different plant extracts used to prepare various metal and semiconductor oxide NPs including zinc oxide, gold, iron oxide, MgO and silver^10–12^ and the others has been described in several publications. Few authors also report the phytosynthesis and degradation potential of SnO_2_ by using different plant extracts^13,14^. While SnO_2_ proficiency in UV absorbance is well-established, the need for materials capable of harnessing visible light has become increasingly imperative, given its prevalence in the solar spectrum. However, extending this capability into the visible light spectrum remains a challenging endeavor. Some researchers doping SnO_2_ with other elements to obtain the visible region absorbance by decreasing the energy gap between conduction band and electron band^15,16^. However others obtained the visible region absorbance spectrum without any metal dopant only by different functional groups present in plant extract^17^. In this context, our research seeks to explore alternative avenues, leveraging the unique properties of plant extracts, particularly those derived from *Croton macrostachyus*. This study embarks on a novel exploration, harnessing the photoactive constituents of *Croton macrostachyus* to unlock absorbance capabilities in the visible light spectrum. To do this we investigated the application of different concentrations of plant extract by taking different ratios and the result shows visible light photocatalytic degradation as-synthesized SnO_2_ nanoparticles. Therefore, in this study, the environmentally friendly production of SnO_2_ NPs utilizing extracts of *Croton macrostachyus* for decomposition of rhodamine B (RhB) and Methylene blue (MB) dye under visible light was reported.

### Experimental procedure and materials used

All the chemicals (stannous tetrachloride pentahydrate (98%) (Molar mass (SnCl_4_, (M = 350.60)), Rhodamine B (RhB), Methylene blue (MB), isopropanol (IPA) and Na_2_EDTA were obtained from Sisco Research Laboratories Pvt. Ltd. In all the experiments, DI water and ethanol were used as a solvent. The chemicals were of analytical quality and employed without additional purification. Green synthesis of tin oxide nanoparticles from tin tetrachloride pentahydrate (SnCl_4_.5H_2_O) and plant extract involves a simple and environmentally friendly method. Here's a concise methodology: the *Croton macrostachyus* plant is chosen which is rich in bioactive compounds (phytochemicals) suitable for nanoparticle synthesis. The plant was extracted using water as a solvent. A tin salt precursor was used as the source of tin ions for NP formation. The plant extract is mixed with the tin salt solution in a controlled environment. The ratio between the plant extract and tin salt concentration can influence the size and stability of the nanoparticles. Heat is applied to maintain specific reaction conditions and to facilitate the reduction of tin ions by phytochemicals available in the plant extract. This reduction leads to the formation of tin oxide nanoparticles. To control the size and stability of the nanoparticles, reaction parameters such as temperature, pH, and reaction time were adjusted. The techniques of centrifugation and filtration are used to separate the synthesized tin oxide nanoparticles from the reaction mixture and washed to remove any impurities or unreacted precursors from the nanoparticles. The obtained nanoparticles were dried under controlled conditions to prevent agglomeration and ensure stability. The synthesized tin oxide nanoparticles were analyzed using characterization techniques like UV–visible spectroscopy, X-ray diffraction (XRD), scanning electron microscopy (SEM), EDX, and Fourier-transform infrared spectroscopy (FTIR) to confirm their size, structure, and composition. The potential applications of the green-synthesized tin oxide nanoparticles was explored and identified as used in visible light photocatalysis. The plant we have used in this report was cultivated in the local area of Nekemte Town, Oromia, Ethiopia. This study complies with relevant international, national, institutional and legislative guidelines.

### Preparation of plant extract

The green leaf of *Croton macrostachyus* was harvested from the Oromia region, East Wollega area, and Guto Gida woreda around Nekemte town. After the leaf was harvested, it was washed with ethanol and dried at room temperature. After drying, the plant was grinded using a grinder in the laboratory. 10 g powder of leaf was taken out and mixed in a borosilicate beaker with 200 ml of DI water and stirred at a temperature of 60 °C, 900 rpm for 4 h. The solution was then filtered using vacuum filters accordingly and stored in the refrigerator for further study.

### Green synthesis of tin-oxide nanoparticles (G-SnO_2_ NPs)

The 3 g of stannous tetrachloride pentahydrate (98%) was dissolved in 100 ml of DI water. To explore the effect of the extract on the properties of SnO_2_ NPs different concentrations of samples were prepared as 1:1, 1:2, 1:3, 1:4, ratios of precursor to the extract following 10 ml:10 ml, 10 ml:20 ml, 10 ml:30 ml, 10 ml:40 ml respectively. Then, the solution was stirred at a temperature of 80 °C for 2 h at a revolution of 700 rpm. The solution was then sonicated for 30 min and cooled overnight. The solution was washed two times with ethanol and centrifuged for 20 min. The solution is dried for 2 h at 200 °C and calcined at 500 °C for 3 h. Subsequently, different concentrations of SnO_2_ NPs were obtained which were used for UV–visible spectroscopy characterization, and for the whole characterizations and applications the 1:4 ratio is selectively used.

### Photodegradation experiments

**Figure 1:**
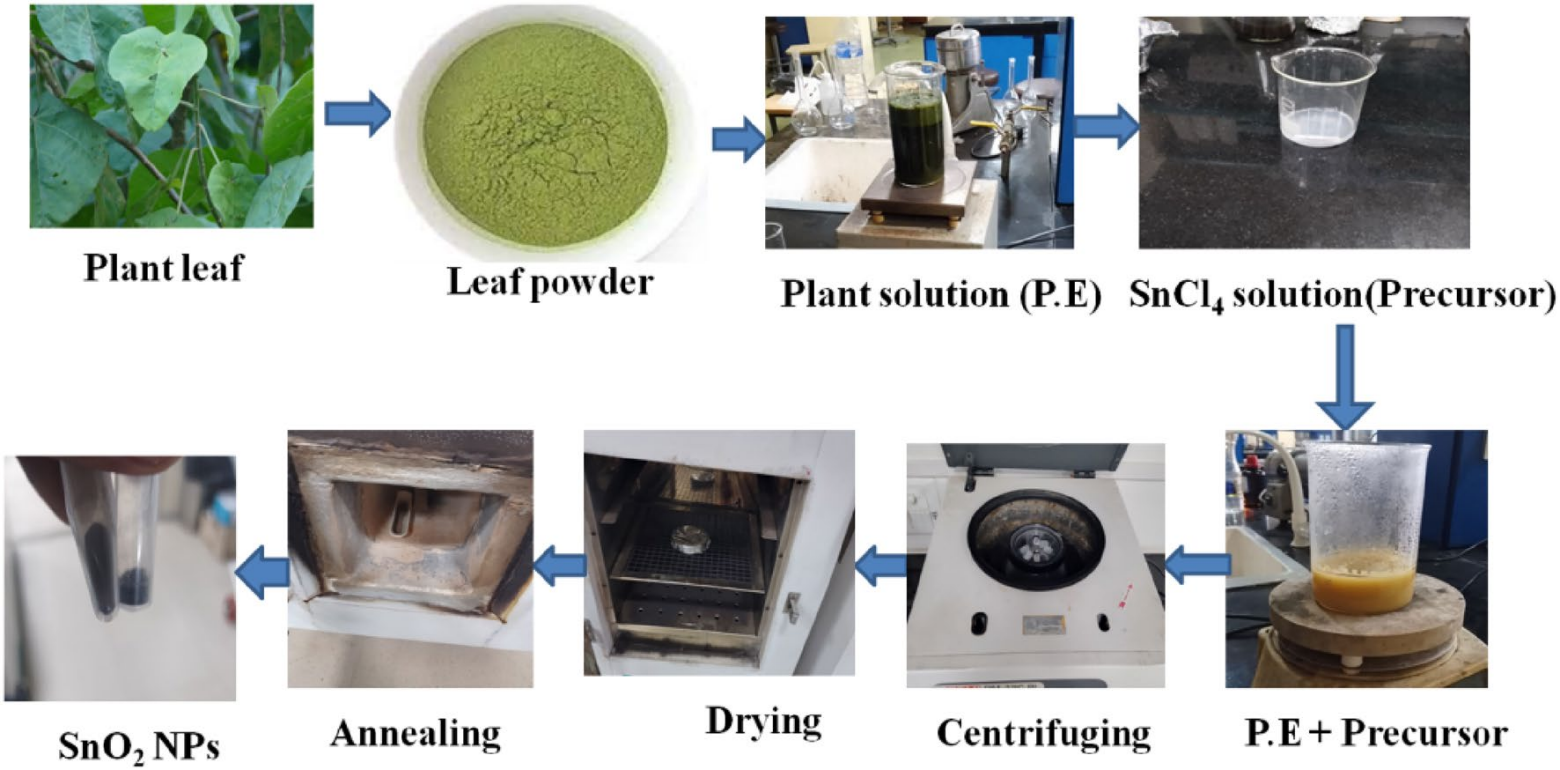
Schematic diagram of Green synthesis route in SnO_2_ NPs.

The degradation potentials of the prepared nanomaterials were studied through the decomposition of rhodamine B (RhB) and Methylene blue (MB) dyes. The solution was prepared for the rhodamine B (RhB) and Methylene Blue (MB) dyes by dissolving (0.1 g/L) of dyes powder in DI water. In this particular research, 50 mg of the metal oxide was treated with an aqueous solution of rhodamine B (RhB) and Methylene blue (MB) in DI water. Ultraviolet Visible (UV–Vis) spectra, observing the change in time of irradiation were occasionally recorded. Schematic diagram of Green synthesis route in SnO2 NPs are depicted in Fig. 1.

## Results and discussion

### UV–Visible spectroscopic studies

**Figure 2:**
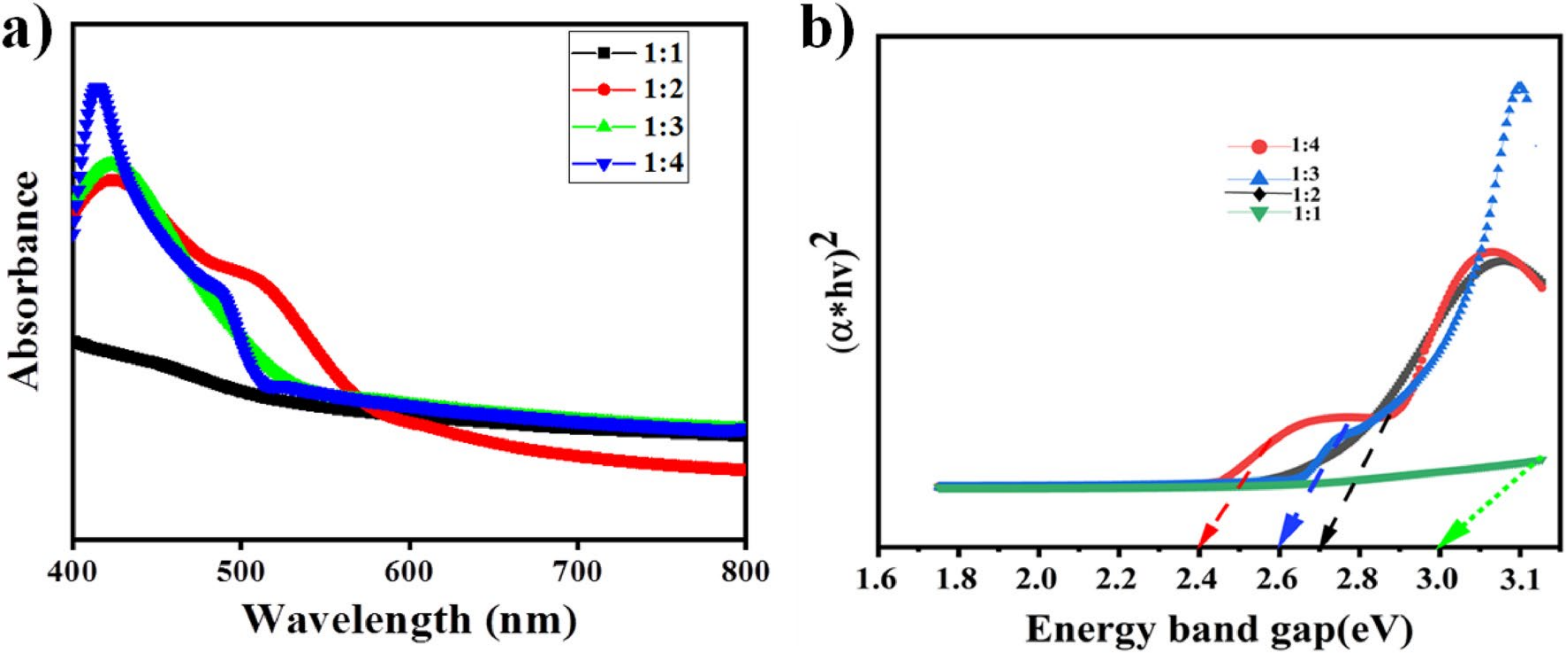
(**a**) UV–Visible spectra of synthesized SnO_2_ nanoparticles and (**b**) their corresponding TAUC’s plots.

The UV–Visible spectroscopy was taken in the wavelength range of 200–800 nm, for the different samples prepared with different ratios of precursor to extract as 1:1, 1:2, 1:3, 1:4 for SnO_2_ NPs. Figure 2a,b shows the maximum absorption peaks of the as-synthesized SnO_2_ NPs for different concentrations and their corresponding Tauc’s plot. From the result one can conclude that as the concentration of the extract increases the properties of the catalyst changes due to electron transfer from the reducing and capping agent present in extract to the precursor and bioreduction happens which further affects the band gap of the synthesized nanoparticles. The band gap (E_g_) of the material, is one of the optical features that demonstrate the semiconductor character of NPs. The optical property of the nanoparticles is obtained from TAUC’s relation below^18^:

1 $$( {\alpha h\nu } )^{2} = K(h\nu - E_{g} )$$

ere $\alpha$, $h$, $\nu$, $E_{g}$, and K are the absorption coefficient, Planck constant, light frequency, band gap energy, and TAUC constant, respectively.

As shown in Fig. 2b below, TAUC’s figure has been used to determine the band gap of semiconductor nanometallic oxides. TAUC numbers are obtained by plotting $$(\alpha h\nu$$)^2^ vs $$h\nu ({\text{energy}}$$$$)$$^17^ and projecting the line onto the x-axis to decide the energy gap values. SnO_2_ NPs has a related assessed band gap values (see Table 1) which shows different functional groups present in extract contribute to the electron transfer, to decrease the energy band gap and this report is agreed with previous report in which the band gap is decreased without any dopant by functional groups present in plant extracts^17–19^. When irradiated with visible light, these band gap values indicate the materials' potential for use in visible light spectrum photocatalysis^20^. The drop in energy gap value of 3.56 eV may also be attributed to several flaws in the crystal structure, such as tin interstitials, oxygen vacancies, or crystal defects. According to certain research, crystal faults expand a new energy level known as Fermi energy levels, which may be the reason why the energy gap between materials narrows. This proposes the involvement of larger particle size and the presence of the Sn–O phase, since the electronic structure and band gap of tin oxide(SnO_2_) are significantly influenced by the particle size, the structural change and particle morphology^21^. The sample's oxygen content is further confirmed by the band gap's narrowing^19^.

**Table 1:** Band gap energy and Particle size studies for SnO_2_ NPs with different concentrations.

S. no	Catalyst ratio	Band gap energy (eV)	Particle size (nm)
1	1:1	3.03	82
2	1:2	2.71	80
3	1:3	2.60	84
4	1:4	2.41	83.8

### FTIR analysis

**Figure 3:**
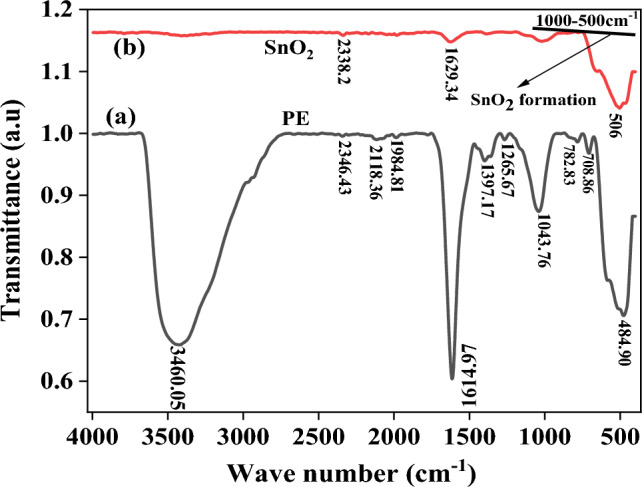
(**a**) FTIR spectra of PE (Plant Extract) and (**b**) reduced biosynthesized SnO_2_ NPs.

The ATR-FTIR spectra of *Croton macrostachyus* extracts along with synthesized SnO_2_ NPs extract are shown in Fig. 3. Figure 3a shows the IR spectra of plant extract which indicate the presence of bio-reduction in plant extract. The absorption pattern at 491.95 cm^−1^ represents the Alkyl and Aryl Halides C–I stretch are associated. The absorption spectra at 1037.6 cm^−1^ describe Alkyl and Aryl Halides S=O group. The peak at 708.86 represents strong C=C bending stretch strongly distributed. The peak at 1614.97 and 1629.34 cm^−1^ corresponds to stretching vibration due to the C=C stretch Alkenes group. The alcohol O–H carboxylic acid intermolecular bonded was present in the absorption at 2346.43, 3460.05 cm^−1^. The peak at 782.83 cm^−1^ represents strong C-H bending 1, 2, 4-trisubstituted cpd. Figure 3b shows the formation of SnO_2_ NPs after the reaction. Here all the functional groups present in plant extract are reduced and only the properties of the required nanoparticle are shown on the graph which indicates the formation of SnO_2_ nanoparticles. The peak at 1629 cm^−1^ represents C=C stretching alkenes di-substituted. The absorption pattern at 506 cm^−1^ shows the Sn–O functional group. Generally, the interpretation of FTIR spectra from the study is well supported by the previous study in which the synthesis of SnO_2_ NPs was performed using extracts of different plants^22^.

### XRD observations

**Figure 4:**
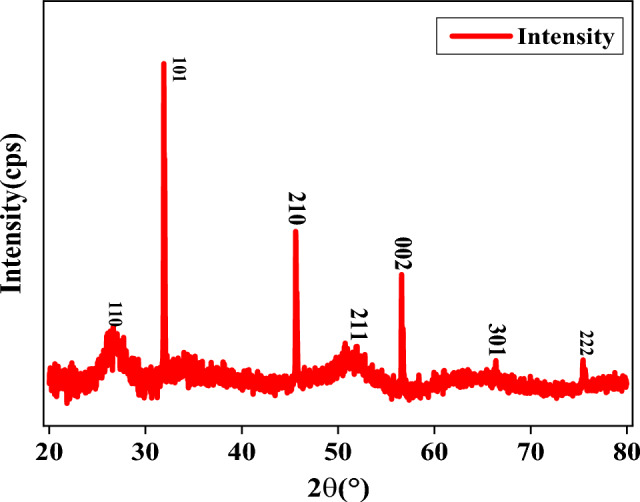
XRD patterns of SnO_2_ NPs from *Croton macrostachyus* leaf extracts.

The XRD diffraction is one of the instruments used to discover the crystalline structure and particle size of the prepared NPs. Figure 4 shows the XRD patterns of all green synthesized SnO_2_ NPs. The peaks with 2θ values at 26.83^∘^, 31.93^∘^, 45.63^∘^, 50.96^∘^, 56.63^∘^, 66.38^∘^ and 75.9^∘^ are associated with the (110), (101), (210), (211), (002), (301) and (222) planes, respectively, indicating the formation of SnO_2_ with spherical structure. This is indexed with the JCPDS card of SnO_2_ nanoparticles (File no: 041-1445). The result obtained in this study agreed with previously reported^18^. The average crystallite size was measured using Debye–Scherer’s Equation^23^ and the average crystallite size (D) was calculated to be 32.18 nm.

Debye–Scherer’s equation is given by:

2 $$D=\frac{0.9\lambda }{\beta {{\cos}}\theta }$$

where $D$ is the crystallite size, $\lambda$ is the wavelength, $\beta$ is the full-width half maximum (FWHM) in radians, and $\theta$ is the Bragg’s angle. The SnO_2_ NPs crystal size calculation using Debye–Scherer’s equation and data from Fig. 4 are listed in Table 2.

**Table 2:** The SnO_2_ NPs crystal size calculation using Debye-Scherer’s equation and data from Fig. 4.

No. of peaks	Planes	Peak position (2$\uptheta$°)	FWHM	Crystal size (nm)	Average (D) nm
1	110	26.82941	2.23142	3.66005352	32.18362565
2	101	31.93453	0.15058	54.87506314	
3	210	45.63661	0.18445	46.72709149	
4	211	50.96011	4.63981	1.891592969	
5	002	56.6333	0.07744	116.5065061	
6	301	66.3838	7.77615	1.219762428	
7	222	75.9038	24.86218	0.405309908	

### Scanning electron microscope (SEM) and EDX analysis

**Figure 5:**
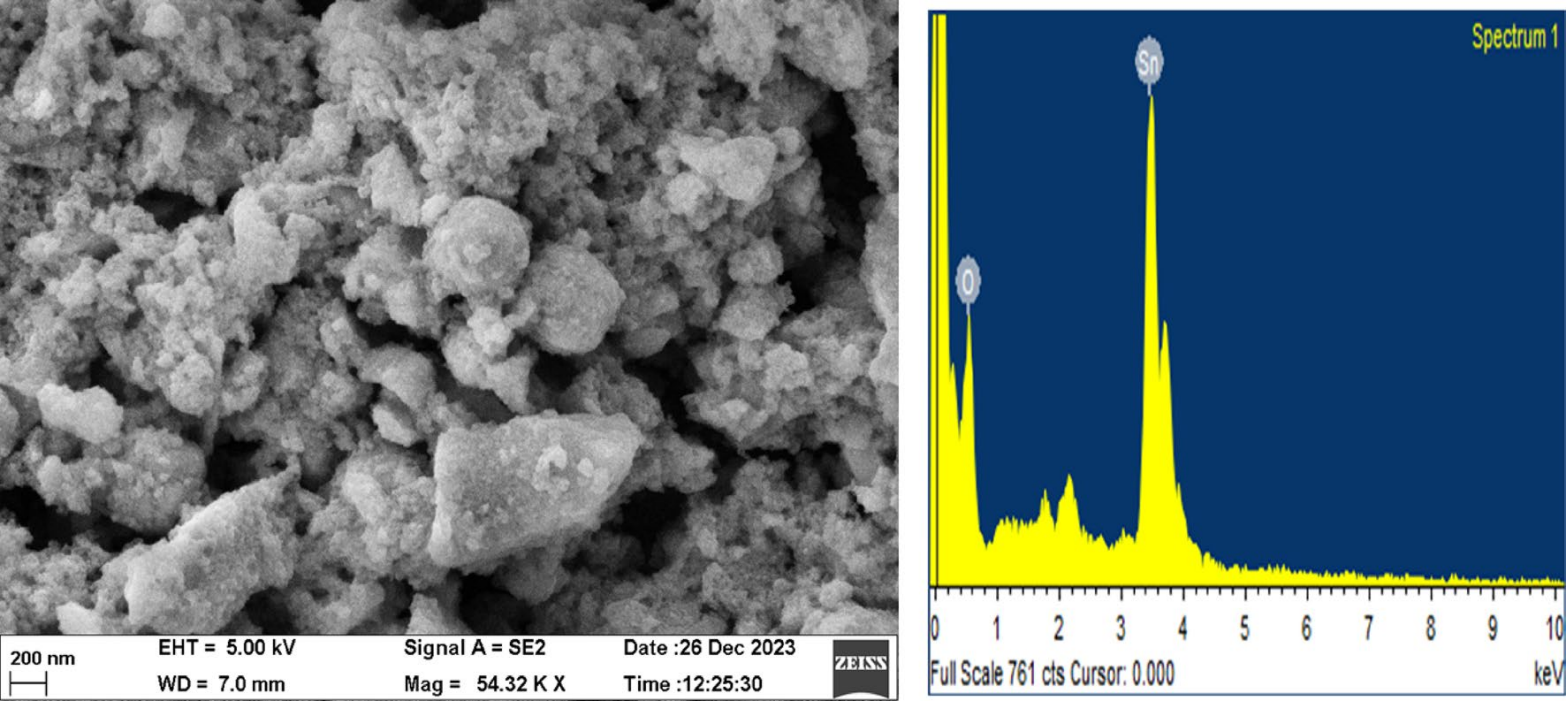
FESEM image and EDX analysis of biosynthesized SnO_2_ NPs extract from *Croton macrostachyus* leaf extracts.

SnO_2_ nanoparticles were prepared from *Croton macrostachyus* leaf extract. The morphological characteristics of these nanoparticles were examined using a field emission scanning electron microscope. The morphology of SnO_2_ nanoparticles in SEM images consists of very fine flakes with tiny agglomerates. The presence of these atoms can attest to the development of the pure SnO_2_ phase^24^. The elements included in the sample are identified using energy-dispersive X-ray spectroscopy (EDAX), which measures the X-rays released from the sample following e-beam excitation. The process relies on an incident electron ejecting an inner shell electron from the sample, ionizing the atoms within. Utilizing EDAX spectrometers, the elemental composition of oxygen and tin was verified (Fig. 5 and Table 3). Energy is shown in kiloparsec (KeV) on the horizontal axis, while the number of X-ray counts is shown on the vertical axis.

**Table 3:** Chemical composition of synthesized SnO_2_ nanoparticles from EDX results.

Element	Weight%	Atomic%
O K	80.22	83.31
Sn L	59.78	16.8612
Totals	100.00	

### X-ray photoelectron spectroscopy (XPS) analysis

**Figure 6:**
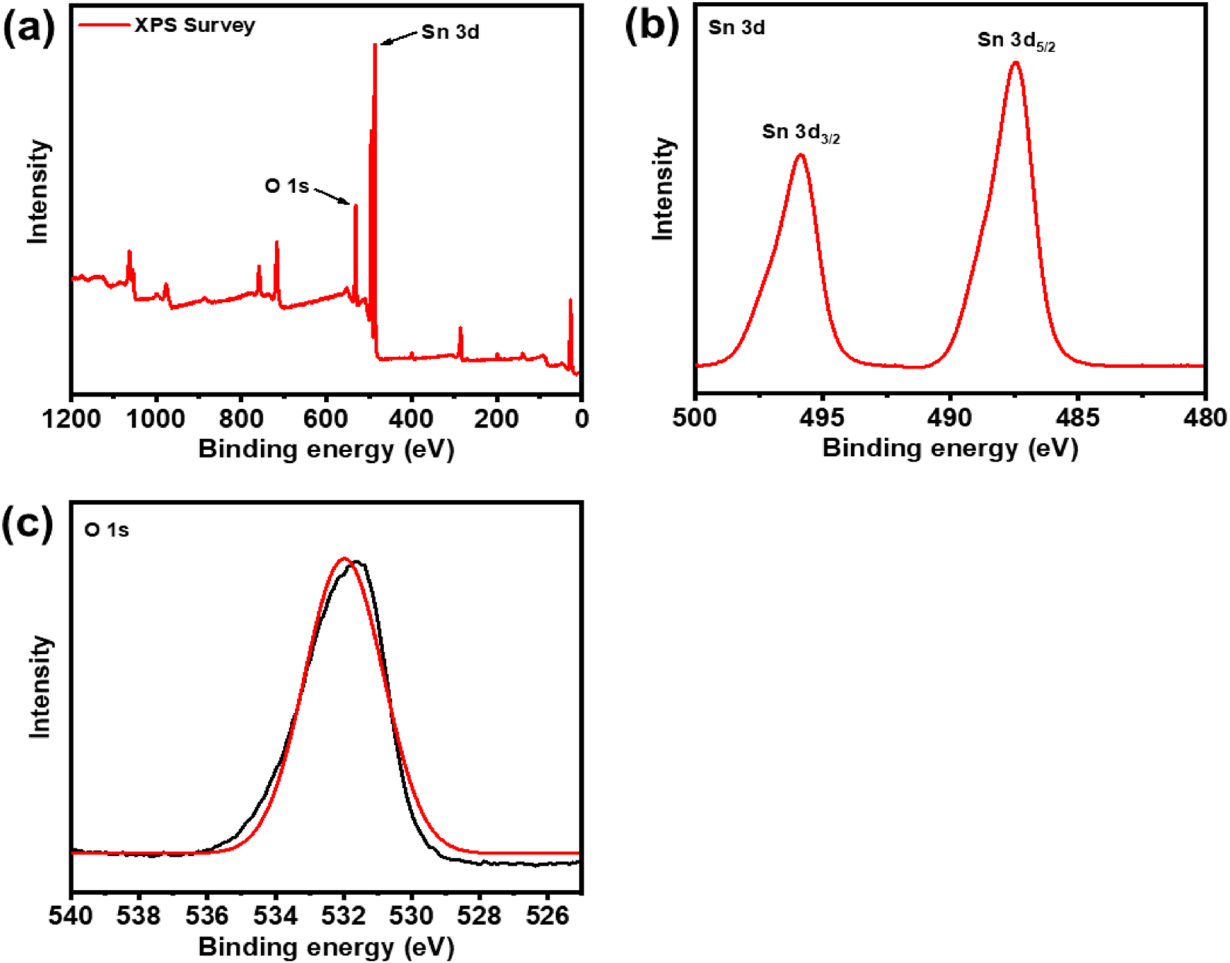
XPS spectra of the as-synthesized SnO_2_ NPs using green method: (**a**) survey scan spectrum, (**b**) Sn 3d, and, (**c**) O 1 s.

To analyze the surface chemistry and compositions of the SnO_2_ nanoparticles, X-ray Photoelectron Spectroscopy (XPS) was employed. Figure 6a displays the overall scan of the samples. Further insights into the chemical makeup are provided in Fig. 6b,c, showcasing the XPS spectra of Sn 3d and O 1 s for the prepared SnO_2_ nanoparticles. Figure 6b reveals doublet peaks corresponding to Sn 3d_5/2_ and Sn 3d_3/2_, providing further characterization of the tin oxidation state. In Fig. 6c, the presence of oxygen is predominantly associated with the surface of SnO_2_, suggesting its bonding with O_2_ ions within the tetragonal structure of Sn^2+^ ions. The non-uniform shape of the O 1 s peak suggests the existence of additional oxygen species on the surface. The XPS analysis of the Sn 3d core-level presents a distinct doublet, suggesting the presence of Sn^4+^ in the SnO_2_ form^25^. Atomic percentage (%) of O 1 s and Sn 3d of the as-synthesized SnO2 NPs was found to be 83.31 and 16.86 respectively.

### Dynamic light scattering (DLS) analysis

**Figure 7:**
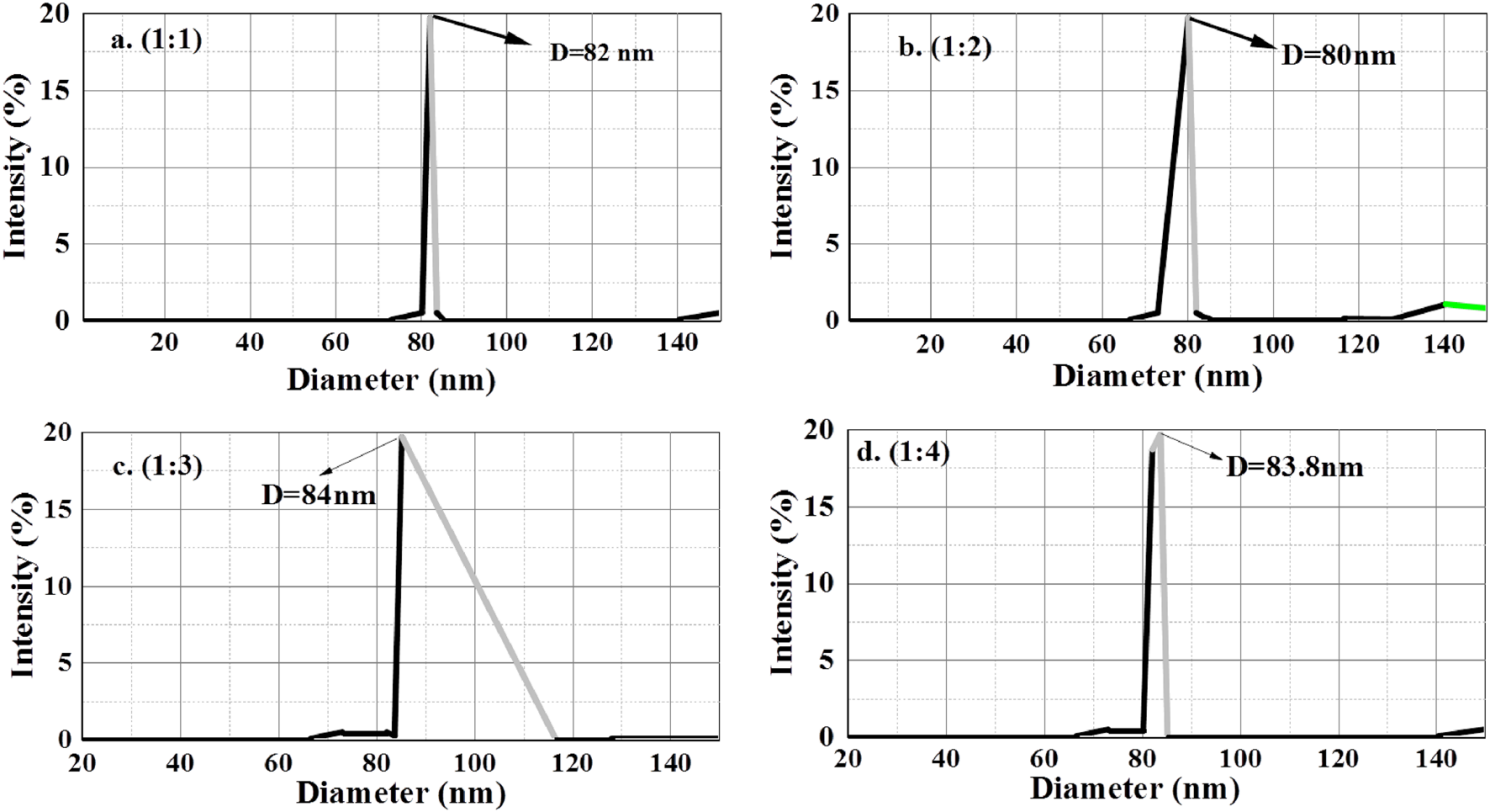
(**a**–**d**) Particle size distribution using DLS for biosynthesized SnO_2_ NPs.

We can only estimate the appropriate crystalline size using Debye Scherrer's equation. Accordingly, one needs either utilize Dynamic Light Scattering (DLS) or transmission electron microscopy (TEM) to obtain a reliable size assessment of nanoparticles despite their size and morphologies. In this work, the DLS approach was used to determine the size distribution and average particle size of the biosynthesized SnO_2_ NPs. Figure 7 illustrates how the SnO_2_ NPs' particle size is generally larger than the size of the crystallite because DLS provides information about the hydrodynamic size of particles, which may differ from their actual physical size, especially for non-spherical or aggregated particles. Additionally, proper data interpretation may involve considering the sample's refractive index, viscosity, and other experimental conditions. Overall, DLS is a powerful technique for characterizing nanoscale particles and colloidal systems, providing valuable insights into their size and stability. Reports of a similar combination employing a chemical approach also exist^10,11^. The crystal size of the different concentrations of the prepared samples were indicated in Fig. 7a–d and it is shown that the average approximate size of the NP is equal for different concentrations of the samples which the difference may not affect the catalytic degradation properties.

### BET analysis/surface area analysis

**Figure 8:**
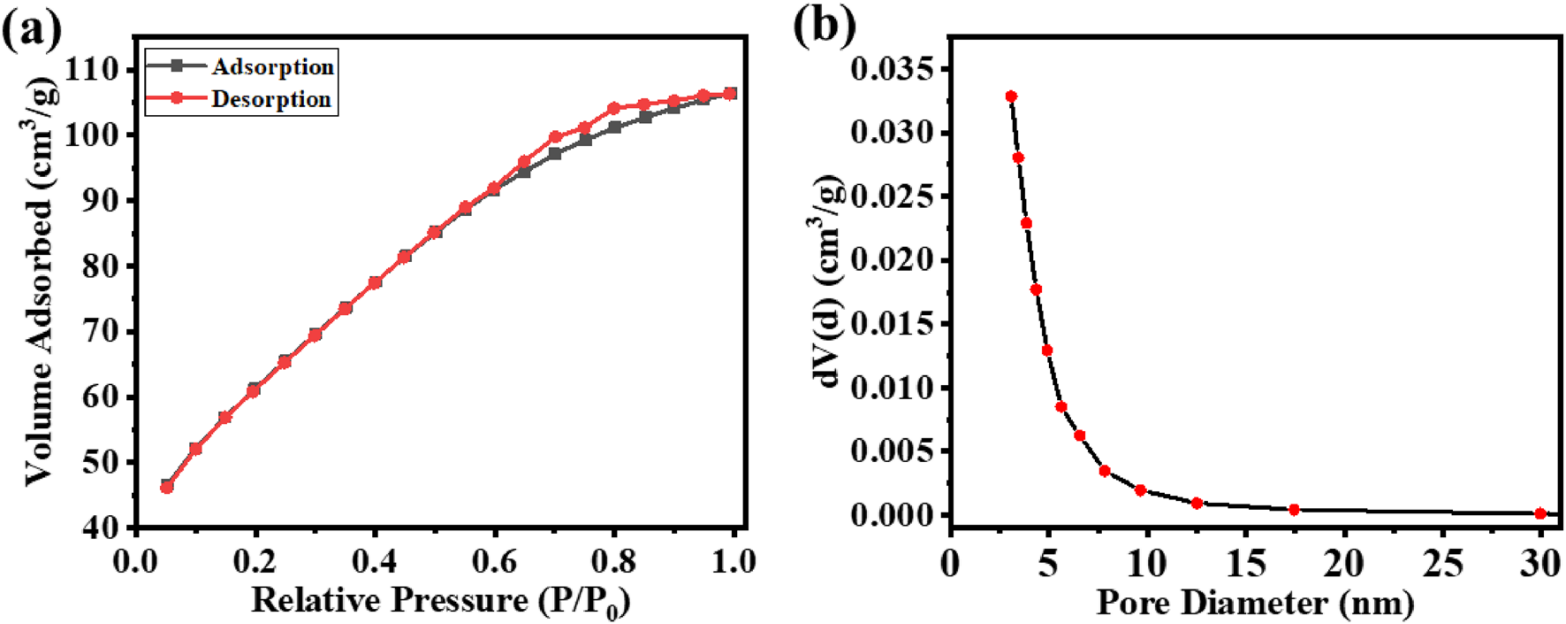
N_2_ adsorption–desorption isotherms of SnO_2_ nanoparticles.

The prepared SnO_2_ NPs surface area and porosity were assessed through a Nitrogen adsorption–desorption experiment conducted at 77 K, as illustrated in Fig. 8. The surface are of the particle is calculated by using Brunauer–Emmett–Teller (BET) method and found to be 212.665 m^2^/g. The BJH (Barret-Joyner-Halenda) method was used to calculate the pore volume and average pore diameter of the as-synthesized SnO_2_ NPs and it is found to be 0.11 cc/g and 3.1 nm, respectively (see Fig. 8a,b).

### Formation of tin oxide (SnO_2_) nanoparticles

Green synthesis typically refers to environmentally friendly methods that aim to minimize the use of hazardous substances and energy. In this case, tin oxide (SnO_2_) nanoparticle is formed from tin (IV) chloride pentahydrate (SnCl_4_⋅5H_2_O), different functional groups in plant extract and deionized water. The pathway for the formation of SnO_2_ nanoparticles is illustrated in Eqs. (3)–(5) below:

**Step 1: Dissociation of SnCl**_**4**_**·5H**_**2**_**O:** SnCl_4_·5H_2_O dissociates in water to release Sn^4+^ ions and Cl^−^ ions:

3 $${\text{SnCl}}_{{4}} \cdot{\text{5H}}_{{2}} {\text{O }} + {\text{H}}_{{2}} {\text{O}} \to {\text{ Sn}}^{{{4} + }} + {\text{ 4Cl}}^{ - } + {\text{ 6H}}_{{2}} {\text{O}}$$

**Step 2: Hydrolysis of Sn4 + ions:** The Sn^4+^ ions react with water molecules to undergo hydrolysis, forming Sn(OH)_4_:

4 $${\text{Sn}}^{{{4} + }} + {\text{ 4H}}_{{2}} {\text{O }} \to {\text{ Sn}}\left( {{\text{OH}}} \right)_{{4}} + {\text{ 4H}}^{ + }$$

**Step 3: Condensation of Sn(OH)**_**4**_**:** The condensation reactions involve the removal of water molecules from Sn(OH)_4_, resulting in the formation of SnO_2_ nanoparticles. The process can be represented as follows:

5 $${\text{nSn}}\left( {{\text{OH}}} \right)_{{4}} \to {\text{ nSnO}}_{{2}} \left( {{\text{nanoparticles}}} \right) \, + {\text{ 4nH}}_{{2}} {\text{O}}$$

The *Croton macrostchayus* organic molecules work as stabilizing sources by binding to SnO_2_ ions, suppressing growth. Consequently, the size of the SnO_2_ NPs determines the percentage of extract that is used^17^. The size and morphology of the SnO_2_ nanoparticles can be influenced by various factors, including the reaction conditions, precursor concentration, and the presence of additives or surfactants.

### Photocatalytic activity studies of prepared SnO_2_ NPs

#### Rhodamine B (RhB) degradation

For degradation purposes we used the 1:4 sample ratios for both RhB and MB dyes. The sample can utilize the visible spectrum of visible light irradiation due to its 2.42 eV band gap after being calcined at 500 °C and this band gap is valuable to use in visible radiation as reported^19^. To assess the SnO_2_ NPs’ ability to degrade effectively under photocatalytic action, rhodamine B (RhB) was used as a contaminant. The photocatalytic activity was supposed to be time-dependent. The photocatalytic experiment was conducted in a stainless steel reactor that was closed. To ensure the solubility of the rhodamine B (RhB) dye at the beginning of the reaction, 50 mg of the photocatalyst samples, a rhodamine B (RhB) from stock solution, and 18 ml of DI water are added and stirred magnetically for 30 min in the dark before light irradiation. After irradiating the solution with visible light, 0.5 mL of the sample was withdrawn every 20 min interval and reported using UV–vis spectroscopy.

**Figure 9:**
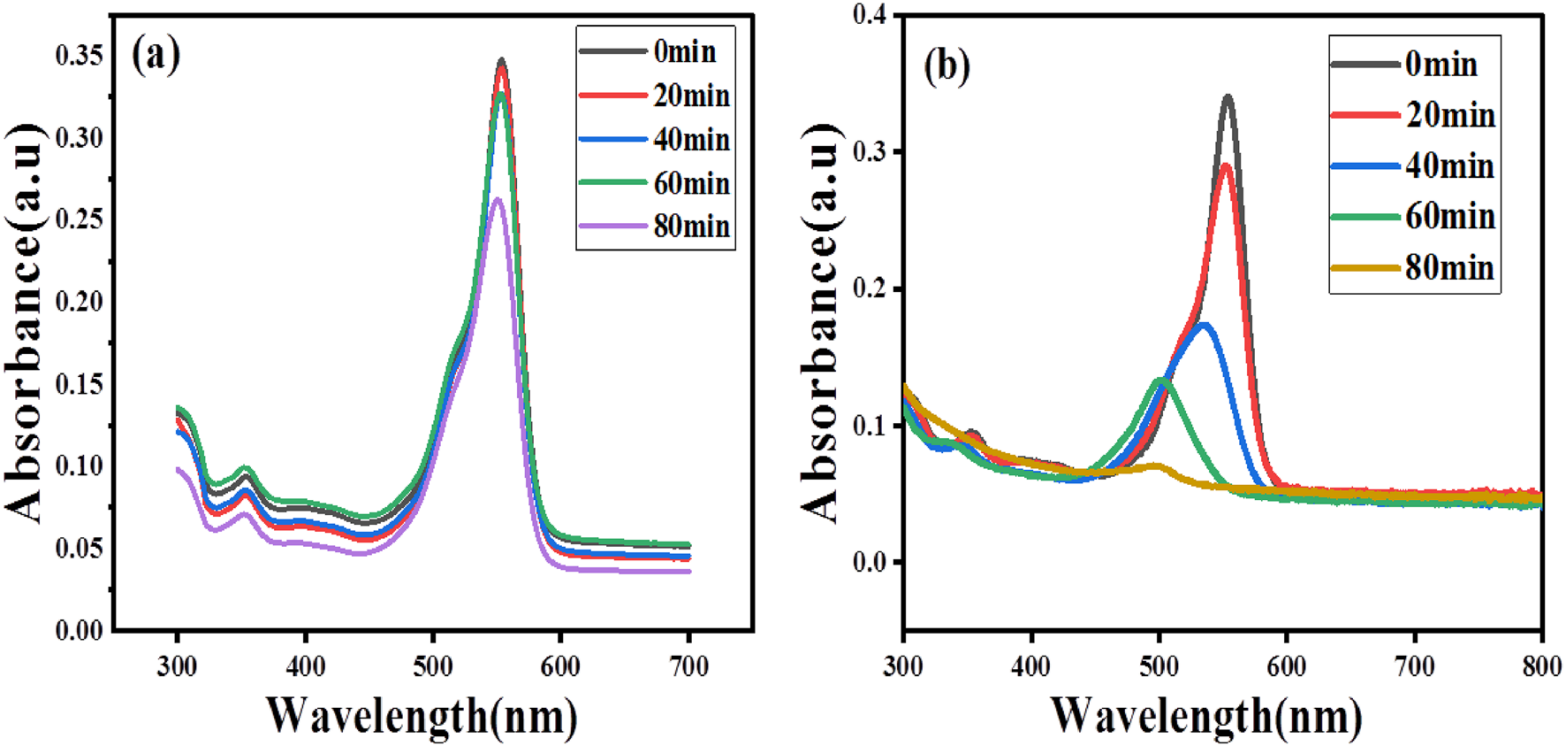
Time-dependent absorption of Rhodamine B (RhB) dye (**a**) without catalyst in 80 min (**b**) with SnO_2_ Catalyst in 80 min.

Rhodamine B (RhB) dye has distinctive absorption spectra at 554 nm, and Fig. 9a,b shows the degradation of RhB following without the addition of catalyst and with the addition of biologically made SnO_2_ NPs in which the degradation is slow in 80 min shows only 7.76% and the absorption intensity steadily declined and vanished in 80 min after the addition of the prepared catalyst shows 86.12% of degradation. Rhodamine B (RhB) dye degrades in an aqueous solution is also evidenced by the visible change in color from pink-red to a colorless solution. From this result, it is concluded that the possible time required for the deprivation of rhodamine B (RhB) dye was 80 min for the prepared solution. This is related to the previous reported literature^26,27^. From the relatively few findings on the visible light photocatalytic performance of SnO_2_, the synthesized sample of SnO_2_ exhibits improved results in the destruction of RhB. The percentage degradation per minute, pseudo-first-order kinetics, and half-life time of degradation were obtained by Eqs. (6)–(8) shown below^28^.

6 $$Degradation \;Efficiency\left(\%\right)=\left(1-\frac{{A}_{t}}{{A}_{o}}\right)\times 100$$

7 $$\ln \frac{{A_{t} }}{{A_{o} }} = - K_{app} t$$

8 $${t}_{1/2}=\frac{0.693}{{K}_{app}}$$

where ${A}_{o}$ and ${A}_{t}$ are the initial concentration and concentration of RhB and MB at the sampling time of ‘t’ respectively, ${K}_{app}$ is the kinetic constant and ${t}_{1/2}$, the half-life time of the dye degradation.

**Figure 10:**
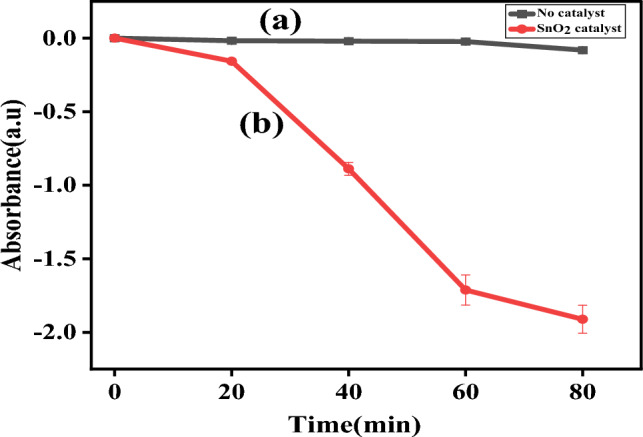
Kinetics of Rhodamine B (RhB) dye photodegradation (**a**) without catalyst (**b**) by addition of bio-synthesized SnO_2_ NPs.

**Figure 11:**
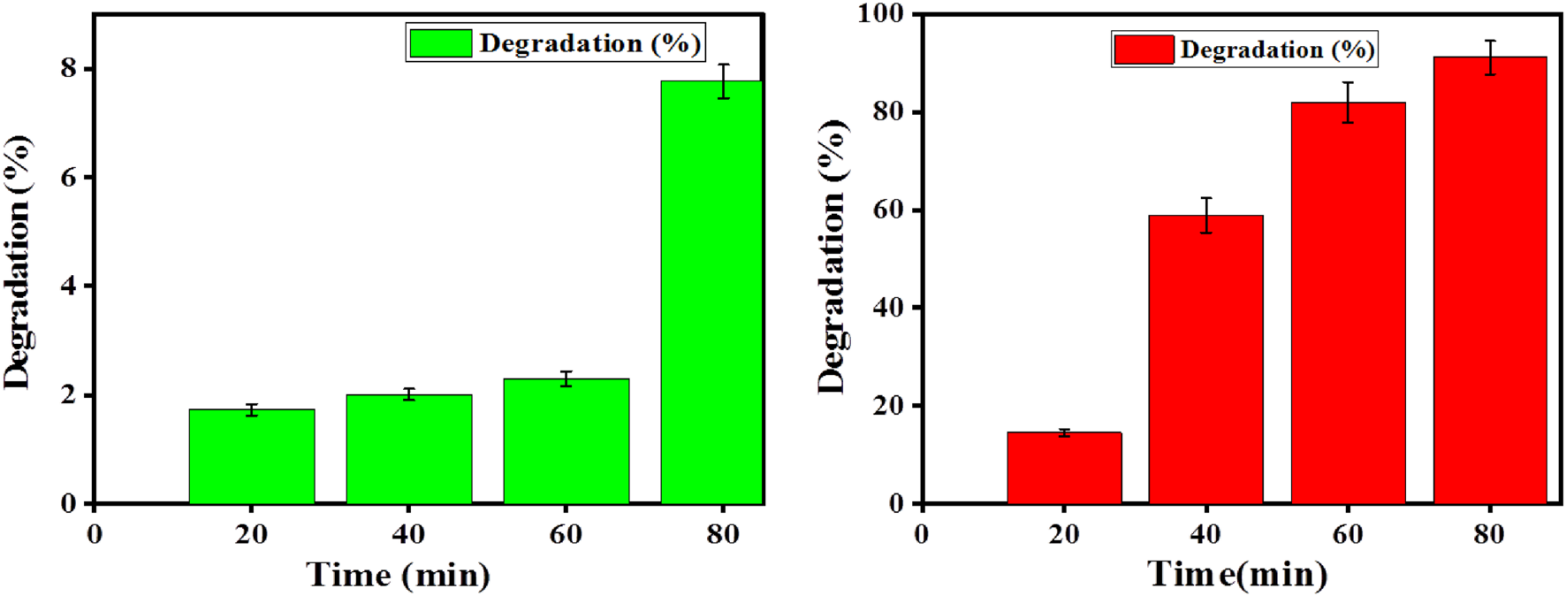
(**a**) Percentage degradation without catalyst and (**b**) Percentage (%) degradation vs synthesized SnO_2_ NPs catalyst dosage for Rhodamine B (RhB) dye.

From the obtained result, the rate constant of degradation per minute was $K$ = 0.02689 (2.689 × 10^−2^ min^−1^), the pseudo-first-order kinetics (Fig. 10) which relies at time increases $lnC/Co$ decreases which resulted in 91.26% (Fig. 11b) of degradation in 80 min and a half life time of degradation was 25.66 min. This value agrees with previous reported literatures^29^. Percentage degradation without catalyst is illustrated in Fig. 11a.

### Methylene blue degradation

The ability of SnO_2_ NPs to break down Methylene blue (MB) in the presence of visible light was assessed using MB as a contaminant. The photocatalytic activity was supposed to be time-dependent. The photocatalytic experiment was conducted in a stainless steel reactor that was closed. To ensure the solubility of the Methylene blue (MB) dye at the beginning of the reaction, 50 mg of the photocatalyst samples, a Methylene blue from stock solution, and 18 ml of DI water are added and stirred magnetically for 30 min in the dark before light irradiation. After irradiating the solution with visible light, 0.5 mL of the sample was withdrawn every 20 min time and reported using UV–vis spectroscopy.

**Figure 12:**
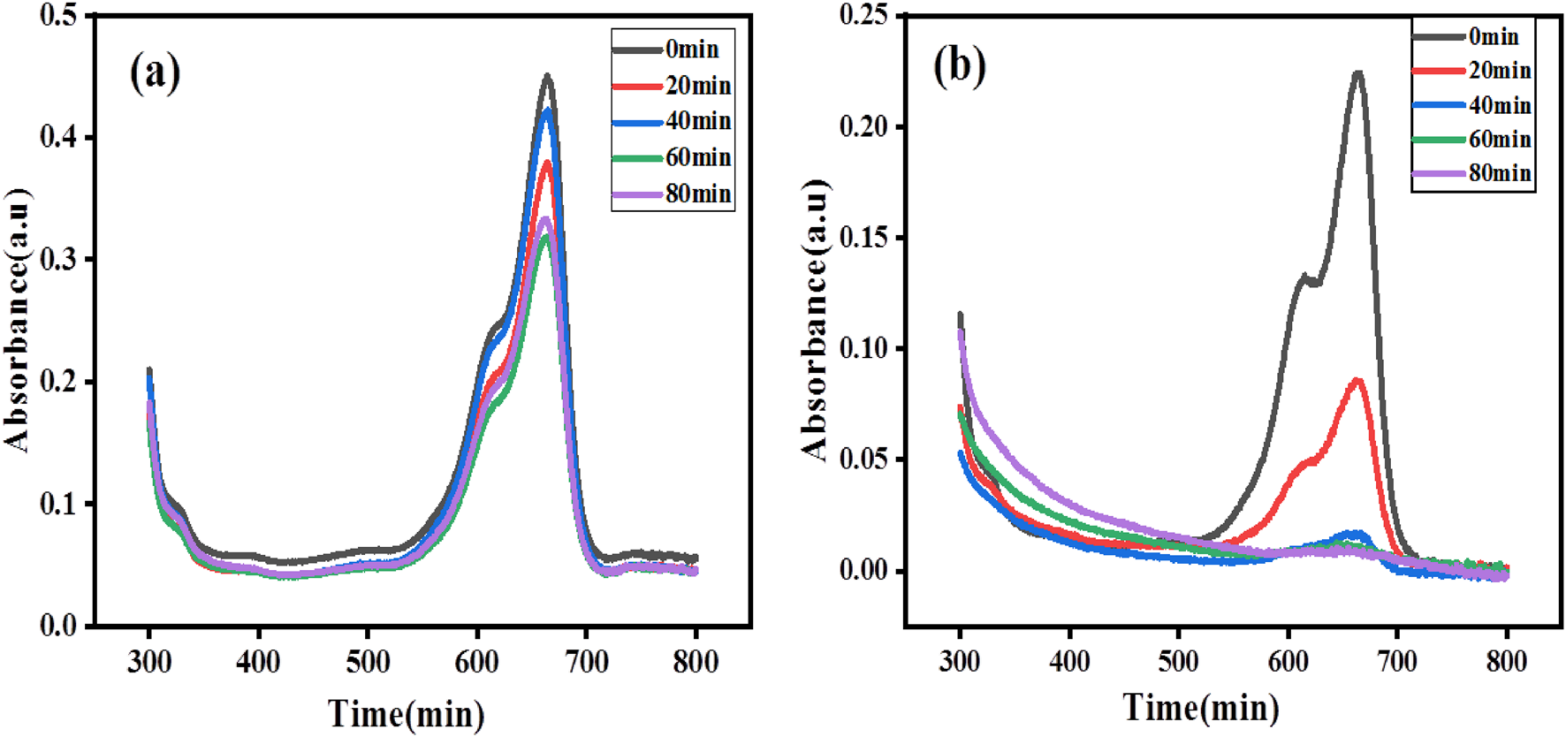
Time-dependent absorption of methylene blue (MB) (**a**) no catalyst (**b**) dye with SnO_2_ catalyst.

The analysis of photocatalytic degradation for the prepared SnO_2_ nanoparticles is shown below for MB under visible light in Fig. 12. MB dye shows typical absorption spectra at 664 nm. The proof of the presence of degradation is indicated by a change in the UV–vis spectra in Fig. 10b. Also the degradation is observed from the color change of blue to colorless during the degradation process. From the spectral changes, the absorbance of the solution along with the time of treatment also reduced. From the graph (Fig. 14b) it is observed that the solution degraded 96.35% after 80 min of visible light exposure. This is because the nanoparticles are smaller and have a narrower band gap, which leads to a higher surface area and improved photocatalytic activity, as shown by earlier studies^17^. For the MB dye, the rate constant of degradation per minute, pseudo-first-order kinetics, and half-life time of degradation was obtained by equation below^28^.

9 $$\ln \frac{{A_{t} }}{{A_{o} }} = - K_{app} t$$

10 $${t}_{1/2}=\frac{0.693}{{K}_{app}}$$

**Figure 13:**
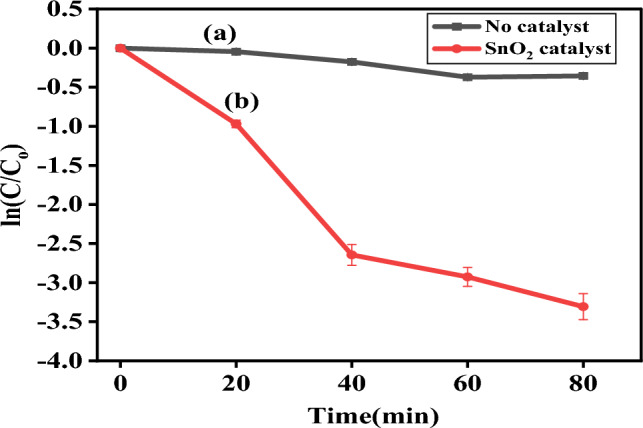
Kinetics of Methylene Blue (MB) dye photodegradation (**a**) without catalyst (**b**) by addition of synthesized SnO_2_ NPs.

**Figure 14:**
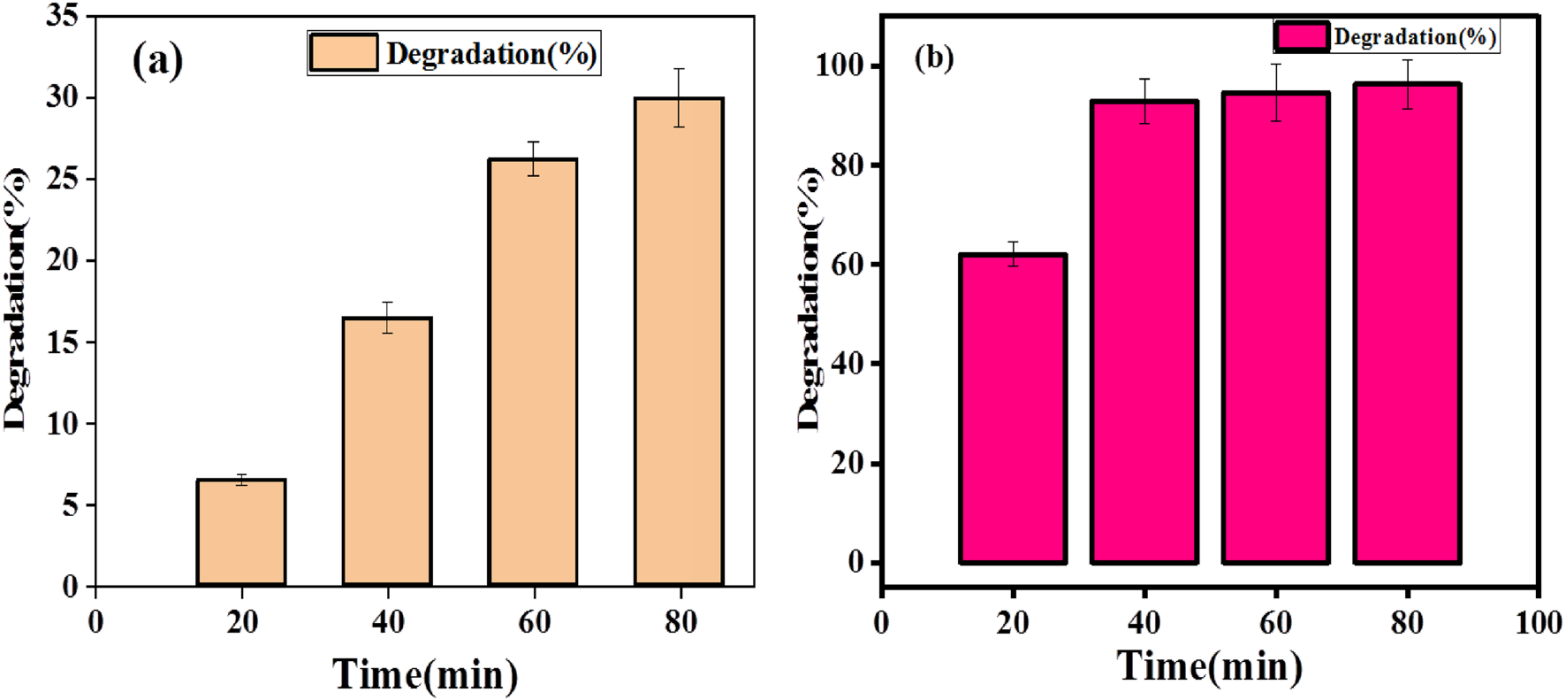
(**a**) Percentage (%) degradation without catalyst (**b**) percentage (%) degradation vs synthesized SnO_2_ NPs catalyst dosage for methylene blue (MB) dye.

From the obtained result, the rate constant of degradation per minute was $K$ = 0.04286 (4.286 × 10^−2^ min^−1^), the pseudo-first order kinetics of degradation before and after the catalyst added (Fig. 13a,b) which relies as on time increases $lnC/Co$ decreases which resulted in 96.35% (Fig. 14b) of degradation in 80 min and a half life time of degradation was 16.1 min. This value agrees with previous reported literatures like^30^. Percentage (%) degradation without catalyst is shown in Fig. 14a.

In general, the percentage of degradation of Methylene blue (MB) dye is faster than that of the Rhodamine B (RhB) dye for the chosen concentration in the result of the study which agrees with the previous report^31,32^.

### Effect of PH on degradation

**Figure 15:**
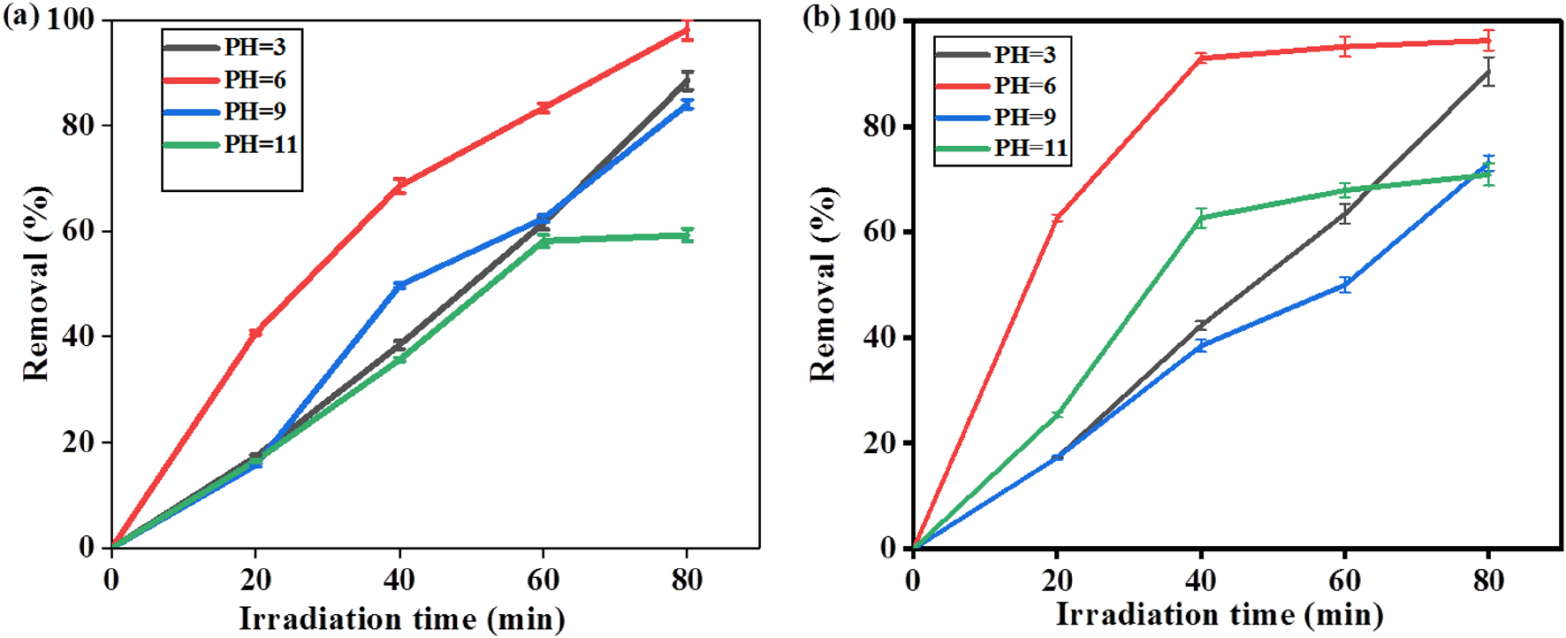
Plot of degradation percentage versus irradiation time for pH dependent on photocatalytic process of SnO_2_ NPs.

Experiments at different pH values were carried out while keeping the catalyst load and dye concentration constant in order to assess the ideal pH for the degradation of methylene blue (MB) and Rhodamine B (RhB) dyes. Protons are released in order to complete the photodegradation. The degradation of RhB and MB for pH 3, 6, 9 and 11 was tested under the irradiation time of 80 min was shown in Fig. 15a,b respectively and it is indicated that maximum PH was observed at PH 6 for both dyes. Beyond this pH, there is less degradation and a thin coating of ions with the opposite charge is drawn to the surface of the nanoparticles due to their surface charge. The catalyst's surface becomes less able to produce hydroxyl radicals as a result of producing more hydroxyl ions, which slows down the dye's degradation.

The strong dye absorption on the catalyst is shown at pH values of 9 and 11 for both dyes, which causes the catalyst's active sites to drastically decrease and produce hydroxyl radicals, which in turn cause the dye's degradation to decrease.

### Comparison of degradation efficiency

Table 4 shows the comparison of degradation efficiency of SnO_2_ NPs with different previously reported study. The comparison shows that SnO_2_ NPs synthesized using the extract of croton macrostachyus is admirable to utilize in the degradation of effluents in water and also they attain improved results than investigations described in the literatures (see Table 4).

**Table 4:** Comparison table of percentage degradation of MB and RhB dye from SnO2 nanoparticles prepared by different methods.

Photocatalyst	Photocatalyst dosage	Pollutant (initial dye concentration)	Photo reactors	Degradation rate	References
SnO_2_	0.25 g	RhB (40 mg L^−1^)	Uvb lamp	65% (180 min)	^14^
SnO_2_	5 mg	RhB (10 mg L^−1^)	UV light	91.7% (190 min)	^29^
SnO_2_ nanostructures	100 mg	RhB (10 mg L^−1^)	UV lamps	62% (240 min)	^33^
SnO_2,_ ZnO	0.1 g	Con-R (10 ppm)	Visible light	70%, 85% (150 min)	^34^
SnO_2_	0.53 g	MB, EBT (100 ppm)	Sun light	90%, 83% (5 h)	^35^
TiO_2_	0.1–0.7 g	RhB (10 mg L^−1^)	UV light	90% (80 min)	^36^
SnO_2_ QDs	0.5 g	RhB (40 mg L^−1^)	UV light	67% (180 min)	^9^
Ag doped ZrO_2_	100 mg	RhB (5 mg L^−1^)	Visible lamp	75% (105 min)	^37^
CTAB-assisted SnO_2_	200 mg	MO (10 mg L^−1^)	Xenon lamp	79% (120 min)	^38^
SnO_2_ NPs	50 mg	MB (0.1 g L^−1^)	Visible light	96.35% (80 min)	This work
SnO_2_ NPs	50 mg	RhB (0.1 g L^−1^)	Visible light	91.26% (80 min)	This work

### Photodegradation in the existence of radical scavengers

**Figure 16:**
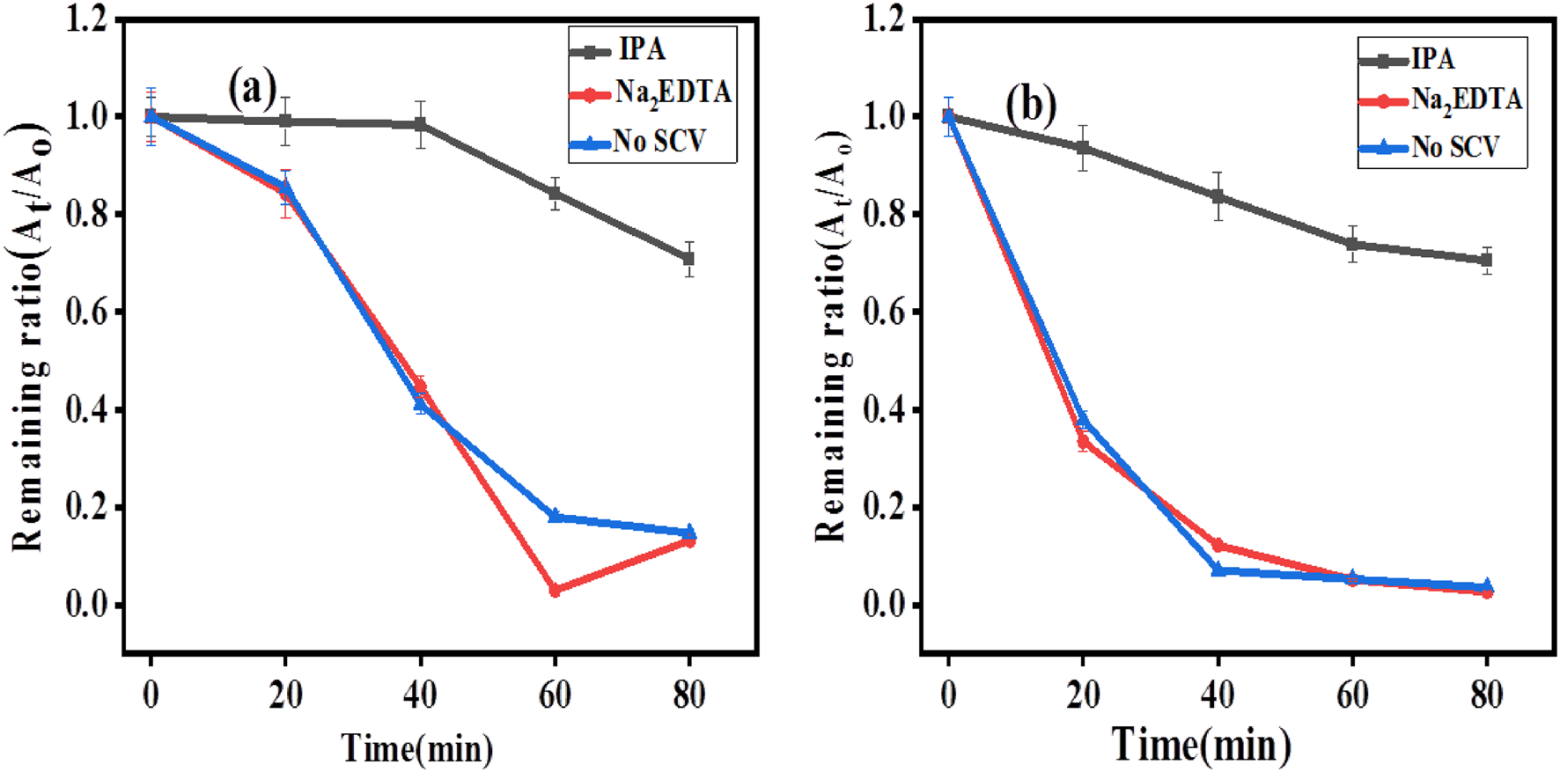
(**a**) The degradation mechanism of RhB and (**b**) MB over biosynthesized SnO_2_ NPs in the presence of isopropanol and Na_2_EDTA radical scavengers.

The primary oxidative species involved in the photodegradation of RhB and MB dyes are identified by radical trapping experiments to further elucidate the potential reaction mechanism of RhB and MB photodegradation by biosynthesized SnO_2_ NPs. In this study, hydroxyl radicals (OH^−^) and holes (h^+^) were scavenged using 3 mM isopropanol and Na_2_EDTA, respectively. As illustrated in Fig. 16a, the addition of isopropanol reduces the photocatalytic degradation efficiency of RhB in the presence of SnO_2_ NPs from 86.12% in the absence of a scavenger to less than 30%. On the other hand, after the addition of Na_2_EDTA, the photodegradation efficiency gradually increased, indicating that the addition of Na_2_EDTA improves the photodegradation of RhB than that of IPA. Therefore, from this result, it is concluded that the IPA restrained the reaction in the case of RhB degradation, and thus electron (e^−^) is predominant in the reaction kinetics. In the case of MB dye Fig. 16b, the addition of isopropanol again restrained the reaction, and the degradation efficiency was reduced to less than 20%. After the addition of Na_2_EDTA, the degradation increases to 97%. The recombination of electrons and holes is therefore inhibited by Na_2_EDTA's role as a hole scavenger. More electrons are easily able to move to the SnO_2_ NP surface, where they react with O_2_ to produce ^_^OH radicals. These findings unequivocally show that the main reactive species in charge of the photocatalytic breakdown of RhB and MB were hydroxyl radicals (^_^OH). The same study has been reported in papers^14,39^ by green and chemical method synthesis respectively for SnO_2_ and TiO_2_ for the photodegradation of RhB and bisphenol A under visible light which validated the result obtained in this paper.

### Photo-oxidation reaction mechanism of dye degradation

The photo-oxidation degradation mechanism for tin oxide nanoparticles involves the interaction of the nanoparticles with light and oxygen, leading to changes in their structure and properties. Tin oxide (SnO_2_) is a semiconductor material commonly used in various applications, including gas sensors, solar cells, and catalysis. When exposed to light and oxygen, tin oxide nanoparticles can undergo photo-oxidation, which is a process where the material reacts with oxygen under the influence of light.

Here is a simplified explanation of the photo-oxidation degradation mechanism for tin oxide nanoparticles: tin oxide nanoparticles absorb photons from light, especially in the UV or visible regions, leading to the excitation of electrons within the material. The absorbed photons create electron–hole pairs within the tin oxide nanoparticles. Electrons are excited to higher energy levels, leaving behind positively charged holes. The excited electrons and holes participate in redox reactions with adsorbed oxygen molecules (O_2_) from the surrounding environment. The oxygen molecules are often adsorbed on the surface of the nanoparticles.

The detailed mechanism can vary depending on the specific conditions, but here is a simplified explanation:

**1**. **Generation of Electron–Hole Pairs:** When tin oxide is exposed to UV/Visible light, electrons in the valence band can be excited to the conduction band, creating electron–hole pairs.

11 $${\text{SnO}}_{{2}} + {\text{ h}}\nu \to {\text{SnO}}_{{2}} * \, + e_{CB}^{ - } + h_{VB}^{ + }$$

where hν represents the energy of absorbed light and SnO_2_* is the excited state of the NPs.

**2. Reactive Oxygen Species (ROS) Formation:** The photogenerated holes (${h}_{VB}$^+^) and electrons (${e}_{CB}$^+^) can react with oxygen and water molecules on the surface to form reactive oxygen species, such as superoxide radicals (O_2_^−^), hydroxyl radicals (OH^−^), and peroxide species.

12 $${\text{O}}_{{2}} + e_{CB}^{ + } \to {\text{O}}_{{2}}^{ - }$$

13 $$h_{VB}^{ + } + {\text{H}}_{2} {\text{O}} \to \cdot {\text{OH}}^{ - } + {\text{H}}^{ + }$$

**3. Oxidation of Organic or Inorganic species:** The generated ROS are highly reactive and can oxidize organic and inorganic species on the surface of tin oxide. This oxidation process leads to the degradation of contaminants.

14 $${\text{Dye (MB or RhB)}} + \cdot {\text{OH}}^{ - } {\text{or O}}_{{2}}^{ \cdot - } \to ({\text{Degradation products}})$$

The overall process involves the generation of electron–hole pairs, the formation of reactive oxygen species, and the subsequent oxidation of contaminants on the tin oxide surface. This mechanism is often exploited in the field of photocatalysis for environmental remediation and degradation of pollutants. Keep in mind that the specific reactions and intermediates involved can vary based on factors such as the type of tin oxide, the presence of co-catalysts, and the nature of the contaminants being degraded.

## Conclusion

In the present study, SnO_2_ NPs were produced by utilizing an eco-friendly synthesis method. The conversion of the bulk tin salt into tin oxide NPs was demonstrated by a change in hue. The FTIR result showed the existence of Sn–O bond peaks at 506 cm^−1^. The band gap calculated from UV–Vis result by TAUC’s relation was recommended as the catalyst for visible light photodegradation of pollutant dyes. In structural analysis, XRD results illustrated that the characteristic peaks of SnO_2_ appear at angles related to the (110), (101), (210), (211), (002), (301), and (222) planes and the crystallite sizes were found to be 32.18 nm. The SEM picture displays agglomerate surface morphology. Our study demonstrated a notable shift from the conventional UV absorbance of SnO_2_ NPs to visible absorbance through the use of *Croton macrostachyus* plant extract. This novel approach opens up exciting possibilities for applications in the visible light spectrum. The practical significance of achieving visible absorbance, with its potential benefits in terms of photocatalytic activities of pollutant dyes under visible light absorbance, suggests that further exploration of this alternative approach is warranted.

When exposed to visible light, rhodamine B and Methylene blue dye undergo photodegradation. Bio-mediated SnO_2_ displayed a maximum degradation efficiency of 86.12% after 80 min of irradiation for Rhodamine B (RhB) and showed 96.35% MB dye after 80 min. From the result, it can be concluded that the photodegradation rate is higher for Methylene Blue when compared to Rhodamine B for equal concentrations of catalyst and dye dosage. From radical scavenging activity, the study revealed that, from electrons and holes the predominant reactive species in the degradation mechanism was hydroxyl radicals (^_^OH). The results show that SnO_2_ NPs have high quality photocatalytic degradation applications. The reason for its high photocatalytic degradation in the visible region for SnO_2_ nanoparticles is its low band gap energy of the prepared NPs and the preparation techniques also affects it.

In general, in this work green methodology was employed to make the semiconductor oxides without the need for a particular environment, and it is an affordable technique for producing nanoparticles at low temperatures. Similar photocatalytic properties to those produced by chemical synthesis can be seen in the metal oxides produced using this approach. Our synthetic process uses metal salts solely, which are used to make metal oxides, and are safe and cheap to utilize. As a result, the semiconductor metal oxides engaged in the present work suggest a green pathway for the degrading processes.

## Data Availability

The datasets used and analyzed during the current study are available from the corresponding author on request.
